# *Callosa* gen. n., a new troglobitic genus from southwest China (Araneae, Linyphiidae)

**DOI:** 10.3897/zookeys.703.13641

**Published:** 2017-09-28

**Authors:** Qingyuan Zhao, Shuqiang Li

**Affiliations:** 1 Institute of Zoology, Chinese Academy of Sciences, Beijing 100101, China; 2 Southeast Asia Biological Diversity Research Institute, Chinese Academy of Sciences, Yezin, Nay Pyi Taw 05282, Myanmar; 3 University of the Chinese Academy of Sciences, Beijing 100049, China

**Keywords:** Asia, cave spider, eyeless, Linyphiinae, morphology, photographs

## Abstract

A new linyphiid genus *Callosa* **gen. n.**, with two new species *Callosa ciliata* **sp. n.** (♂♀, type species) and *Callosa baiseensis* **sp. n.** (♂♀), from southwest China are described. Detailed description of genitalic characters and somatic features is provided, as well as light microscopy and SEM micrographs of each species. *Callosa* **gen. n.** was found in caves in Yunnan and Guangxi, and its copulatory organs are similar to those of *Bathyphantes* and *Porrhomma*, but differ greatly in details. The monophyly and placement of *Callosa* **gen. n.** are supported by the results of molecular analysis.

## Introduction

In previous collecting work conducted in caves in southwest China, a considerable number of troglobitic spider species belonging to Nesticidae, Leptonetidae, Telemidae, and Pholcidae were found, but Linyphiidae were seldom encountered. Due to insufficient efforts in taxonomy, no more than 100 linyphiid species have been reported from there, and only one of them was found in caves. Here a new linyphiid genus collected in caves from southwest China is described, whose copulatory organs identify it as a genus of . It has obvious somatic characters of real cave dwellers, indicating its long-term underground evolutionary history. In order to test its placement in suggested by morphological characters, an additional molecular analysis based on newly sequenced DNA data of the two species and sequences available from GenBank was conducted.

## Materials and methods

Specimens were studied using a LEICA M205 C stereomicroscope. Further details were examined under a BX51 compound microscope. Copulatory organs were examined after being dissected from the spiders’ bodies. Left male palps were used, except as otherwise indicated. Female epigynes and vulvae were removed and treated in warm potassium hydroxide (KOH) water solution before study. All embolic divisions, epigynes and vulvae were photographed after being embedded in gum arabic. Photos were taken with an Olympus c7070 wide zoom digital camera (7.1 megapixels) mounted on an Olympus BX51 compound microscope. Images from multiple focal planes were combined using Helicon Focus (version 3.10) image stacking software. All measurements are given in millimeters. Eye diameters were measured at their widest extent. Leg measurements are shown as: total length (femur, patella, tibia, metatarsus, tarsus). The terminology of copulatory organs follows Saaristo (1995), Tanasevitch (2014).

SEM images were taken using the FEI Quanta 450 at the Institute of Zoology, Chinese Academy of Sciences. Specimens for SEM examination were critical point dried and sputter coated with gold-palladium. Specimens were mounted on copper pedestals using double-sided adhesive tape.

The tibial spine formula, which expresses the number of dorsal tibial spines on each of legs I to IV, is given for species in which it differs from the type species of the genus. The patellar spine formula is given only if it differs from the most common one (1-1-1-1).

All type specimens are deposited in the Institute of Zoology, Chinese Academy of Sciences in Beijing (**IZCAS**), except as otherwise indicated.

Abbreviations used in the text and figures are given below. References to figures in cited papers are noted in lowercase type (fig.).

### Male palp

**CV** convector

**DSA** distal suprategular apophysis

**E** embolus

**MM** median membrane

**PC** paracymbium

**PT** protegulum

**ST** subtegulum

**T** tegulum

### Epigyne

**A** atrium

**CF** copulatory furrows

**CO** copulatory opening

**DP** dorsal plate

**P** parmula

**R** receptacle

**SO** socket

**VP** ventral plate

### Somatic morphology

**ALE** anterior lateral eye

**ALS** anterior lateral spinneret

**AME** anterior median eye

**CY** cylindrical gland spigot

**PLE** posterior lateral eye

**PLS** posterior lateral spinneret

**PME** posterior median eye

**PMS** posterior median spinneret

### Phylogenetic analysis

Analysis conducted here is partially based on the data matrix of Arnedo et al. (2009). A few taxa were taken out, and more taxa of Linyphiinae downloaded from GenBank were added to reconstruct phylogeny. A total of 66 taxa were included for the final test. Partial fragments of the mitochondrial genes cytochrome *c* oxidase subunit I (COI), 16SrRNA (16S) and the nuclear genes Histone 3 (H3), 18SrRNA (18S) were amplified and sequenced for *Callosa ciliata* sp. n. and *C. baiseensis* sp. n. following the procedure in Arnedo et al. (2009). Sequences for each gene were edited in Bioedit (Hall 1999), and aligned in MAFFT (http//mafft.cbrc.jp/alignment/server/). Bayesian inference was performed in MrBayes 3.1.2 (Ronquist and Huelsenbeck 2003) using parameters selected by jModelTest (Posada 2008). The Markov chains were sampled every 1000 generations for 2 million generations, with the first 25% of sampled trees discarded as burn-in. Taxonomic and sequence information of the used taxa are presented in Table 1.

Bayesian inference based on four genes yielded a similar phylogenetic tree to Arnedo’s (Arnedo et al. 2009) and Sun’s (Sun et al. 2014). The *Callosa* gen. n. species belong to as indicated by the cladogram (Fig. 10).

**Table 1.** DNA data information of species included in the phylogenetic analysis

Family	Genus	Species	16S	18S	COI	H3
Pimoidae	*Pimoa*	sp. X131	AY230940	AY230893	AY231025	AY230985
Linyphiidae	*Agyneta*	*ramosa*	FJ838670	FJ838694	FJ838648	FJ838740
	*Anguliphantes*	*nasus*	JN816483	JN816703	JN817115	
	*Australolinyphia*	*remota*	FJ838671	FJ838695	FJ838649	FJ838741
	*Bathyphantes*	*floralis*	GU338604	GU338465	GU338659	
	*Bathyphantes*	*gracilis*	FJ838672	FJ838696	FJ838650	FJ838742
	*Bolyphantes*	*alticeps*	AY078660	AY078667	AY078691	AY078700
	*Callosa* gen. n.	*baiseensis* sp. n.	MF095861	MF095862	MF095863	MF095864
	*Callosa* gen. n.	*ciliata* sp. n.	MF095865		MF095866	MF095867
	*Centromerus*	*trilobus*	GU338599	GU338468	GU338656	
	*Dicymbium*	*sinofacetum*	GU338614	GU338487	GU338665	
	*Diplocentria*	*bidentata*	GU338629	GU338494	GU338688	
	*Diplocephalus*	*cristatus*	GU338637	GU338490	GU338696	
	*Diplostyla*	*concolor*	FJ838673	FJ838697	FJ838651	FJ838743
	*Doenitzius*	*pruvus*	GU338632	GU338474	GU338691	
	*Drapetisca*	*socialis*	FJ838674	FJ838698	FJ838652	FJ838744
	*Dubiaranea*	*aysenensis*	FJ838675	FJ838699	FJ838653	FJ838745
	*Dubiaranea*	*distincta*	GU338624	GU338459	GU338648	
	*Dubiaranea*	*propinquua*	GU338627	GU338460	GU338675	
	*Erigone*	*prominens*		GU338539	GU338679	
	*Eskovina*	*clava*	JN816489	JN816710	JN817122	
	*Floronia*	*bucculenta*	FJ838676	FJ838700	FJ838654	FJ838746
	*Frontinella*	*communis*	GU338628	GU338517		
	*Gnathonarium*	*dentatum*	GU338593	GU338477	GU338651	
	*Haplinis*	*diloris*	FJ838680	FJ838704	FJ838657	FJ838750
	*Helophora*	*insignis*	FJ838681	FJ838705	FJ838658	FJ838751
	*Himalaphantes*	*azumiensis*		GU338522	GU338677	
	*Hylyphantes*	sp. 'irellus'	GU338618	GU338481	GU338668	
	*Kaestneria*	*pullata*	KT003126	KT002937	KT002739	KT002838
	*Labulla*	*thoracica*	AY078662	AY078674	AY078694	AY078707
	*Laetesia*	sp. MAA-20099	FJ838682	FJ838706	FJ838659	FJ838752
	*Lepthyphantes*	sp. 17 SL-2010	GU338610	GU338509	GU338664	
	*Linyphia*	*triangularis*	AY078664	AY078668	AY078693	AY078702
	*Microlinyphia*	*dana*	AY078665	AY078677	AY078690	
	*Microneta*	*viaria*	FJ838684	FJ838708	FJ838661	FJ838754
	*Moebelia*	*rectangula*	GU338591	GU338485		
	*Neriene*	*albolimbata*	JN816480	JN816700	JN817112	
	*Neriene*	*clathrata*	JN816478	JN816698	JN817110	
	*Neriene*	*emphana*	JN816474	JN816694	JN817106	
	*Neriene*	*japonica*	GU338633	GU338462	GU338692	
	*Neriene*	*longipedella*	JN816476	JN816696	JN817108	
	*Neriene*	*nigripectoris*	JN816481	JN816701	JN817113	
	*Neriene*	*oidedicata*	JN816479	JN816699	DQ396860	
Linyphiidae	*Neriene*	*radiata*	AY078710	AY078670	AY078696	AY078709
	*Neriene*	*variabilis*	AY078711	AY078669	AY078699	AY078706
	*Nippononeta*	*kantonis*	GU338634	GU338471	GU338693	
	*Novafroneta*	*vulgaris*	FJ838686	FJ838710	FJ838663	FJ838756
	*Oedothorax*	*apicatus*	FJ838687	FJ838711	FJ838664	FJ838757
	*Orsonwelles*	*malus*	AY078737	AY078676	AY078697	AY078708
	*Orsonwelles*	*polites*	AY078725	AY078671	AY078755	AY078701
	*Pacifiphantes*	*zakharovi*	KT003159	KT002971	KT002771	KT002872
	*Paikiniana*	sp. 8 SL-2010	GU338617	GU338495	GU338647	
	*Parameioneta*	*bilobata*	GU338605	GU338503	GU338660	
	*Parasisis*	sp. 27 SL-2010	GU338592	GU338500	GU338650	
	*Pityohyphantes*	*costatus*	AY078666	AY078675	AY078695	
	*Pocobletus*	sp. MAA-2009	FJ838689	FJ838713	FJ838665	FJ838759
	*Porrhomma*	*montanum*	JN816486	JN816706	JN817118	
	*Porrhomma*	sp. 24 SL-2010	GU338607	GU338466	GU338661	
	*Pseudafroneta*	*incerta*	FJ838690	FJ838714	FJ838666	FJ838760
	*Sisicottus*	*montanus*	GU338625	GU338479	GU338673	
	*Solenysa*	sp. 14 SL-2010	GU338616	GU338506	GU338667	
	*Sphecozone*	*bicolor*	GU338622	GU338496	GU338671	
	*Stemonyphantes*	*lineatus*	FJ838691	FJ838715	FJ838667	FJ838761
	*Tenuiphantes*	*tenuis*	FJ838693	FJ838716	FJ838669	FJ838763
	*Walckenaeria*	*clavicornis*	GU338596	GU338483		
	*Walckenaeria*	*keikoae*	GU338636	GU338484	GU338695	

## Taxonomy

### Family Linyphiidae Blackwall, 1859

#### Genus Callosa gen. n.

Taxon classificationAnimaliaAraneaeLinyphiidae

http://zoobank.org/4EC11D86-CC7A-4467-8AB6-83356A928616

##### Type species.

*Callosa ciliata* sp. n.

##### Etymology.

The generic name is an arbitrary combination of letters. Gender is feminine.

##### Diagnosis.

**Figure 1.**
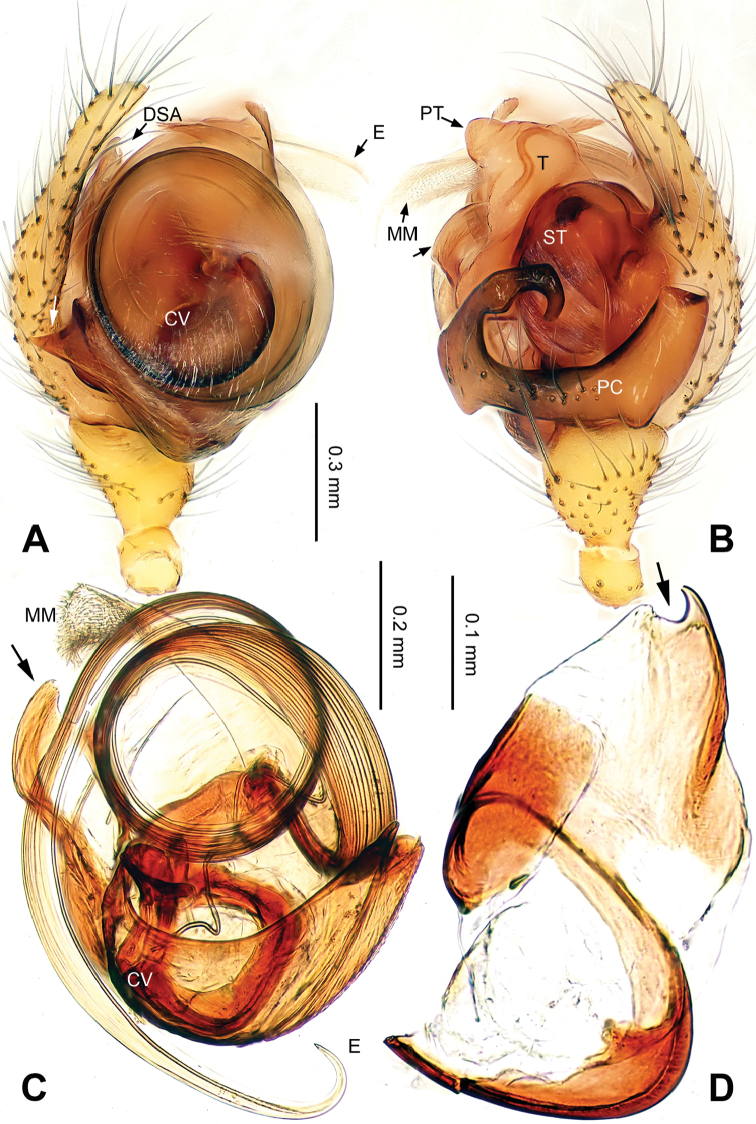
*Callosa ciliata* sp. n., male holotype. **A** Palp, prolateral view **B** Palp, retrolateral view **C** Embolic division, retrolateral view **D** Distal suprategular apophysis, retrolateral view. Scale bars: **B** as **A**.

The copulatory organs in this genus clearly resemble those in , but differ from the similar genera by: embolus in *Callosa* gen. n. is long and forms one big loop (Figs 1A, 5A), neither a short and curved one as in *Porrhomma* Simon, 1884, *Diplostyla* Emerton, 1882, *Pacifiphantes* Eskov & Marusik, 1994 (Roberts 1987: figs 58a–e, 59a–e; Eskov and Marusik 1994: fig. 42), nor an apically coiled one as in most *Bathyphantes* Menge, 1866 (Roberts 1987: fig. 70a–e); the embolus in *Bathyphantes approximatus* (O. Pickard-Cambridge, 1871) is longer and slimmer, forming more than 2 loops (Ivie 1969: fig. 102); *Microbathyphantes* Helsdingen, 1985 has coiled, whip-like, and fully exposed embolus (Tu and Li 2006: fig. 2C), unlike the one enveloped in a membranous plate of the convector in *Callosa* gen. n. The epigyne in *Callosa* gen. n. is distinguished by its long, spiraling copulatory furrows and the presence of a septum (Figs 3C, 7C); the receptacles are situated farther from atrium in most *Bathyphantes* species, furrows are not in double-helix; *Kaestneria* Wiehle, 1956 and *Pacifiphantes* have shorter copulatory furrows, which fold or curve (Slowik and Blagoev 2012: fig. 6); the copulatory furrows in *Microbathyphantes* make only half a turn.

##### Description.

**Figure 2.**
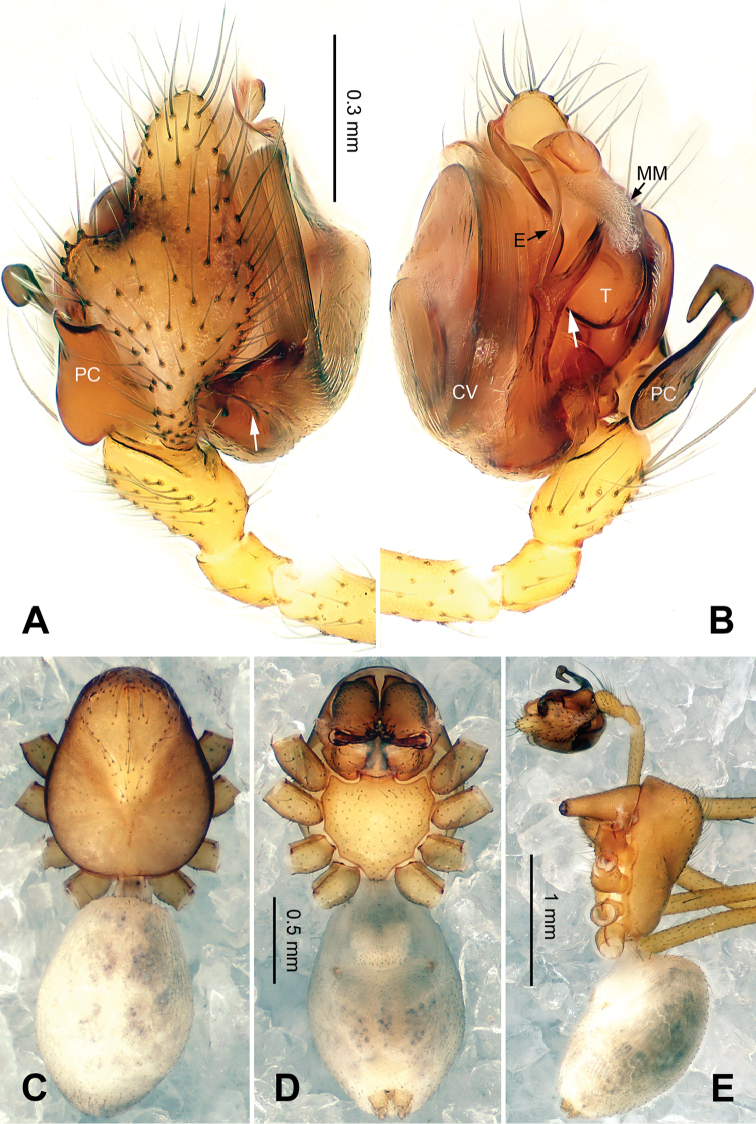
*Callosa ciliata* sp. n., male holotype. **A** Palp, dorsal view **B** Palp, ventral view **C** Habitus, dorsal view **D** Habitus, ventral view **E** Habitus, lateral view. Scale bars: **B** as **A**; **C** as **D**.

**Figure 3.**
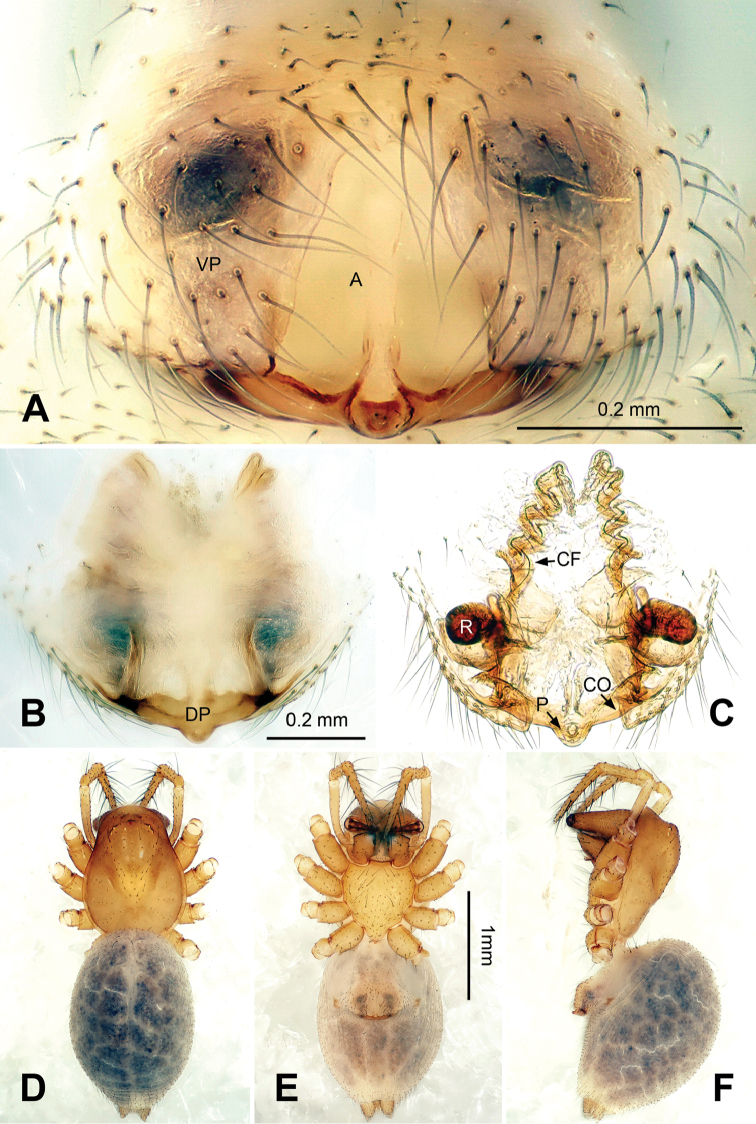
*Callosa ciliata* sp. n., female paratype. **A** Epigyne, ventral view **B** Epigyne, dorsal view **C** Vulva, dorsal view **D** Habitus, dorsal view **E** Habitus, ventral view **F** Habitus lateral view. Scale bars: **C** as **B**; **D, F** as **E**.

Median size, 2.5‒2.8. Chelicerae with three promarginal, and four retromarginal teeth. AME completely lost, PME reduced to small unpigmented spots, ALE and PLE highly reduced (Figs 2C, 2E, 3D, 3F, 6C, 6E, 7D, 7F); ocular area with several rows of short setae (Figs 2C, 6C). Carapace length/leg I 0.13– 0.15. Coxae IV separated by their diameter. Chaetotaxy: 2-2-2-2. TmI 0.15–0.20, TmIV absent. Leg formula I-II-IV-III. Legs yellow without obvious patterns.

**Figure 4.**
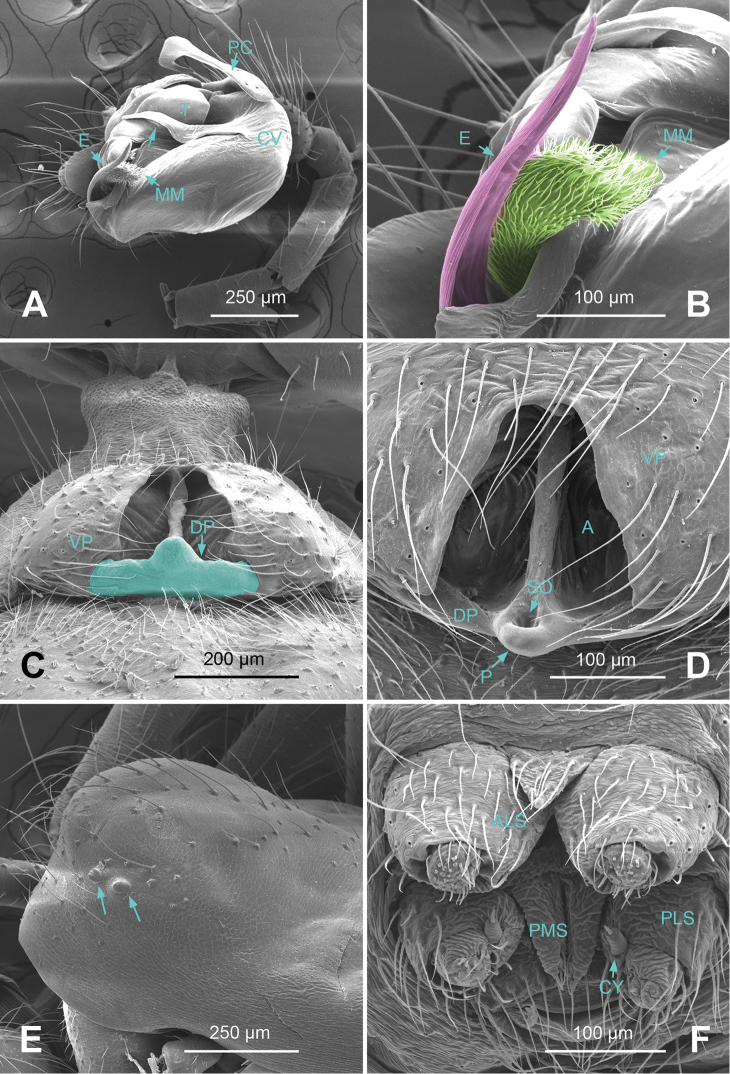
*Callosa ciliata* sp. n., SEM of a male paratype and a female paratype. **A** Palp of male paratype, ventral view **B** Detail showing embolus and embolic membrane of palp **C** Epigyne of female paratype, ventral view **D** Detail showing parmula of epigyne **E** Anterior lateral eye and posterior lateral eye of male paratype **F** Spinnerets of female paratype.

**Figure 5.**
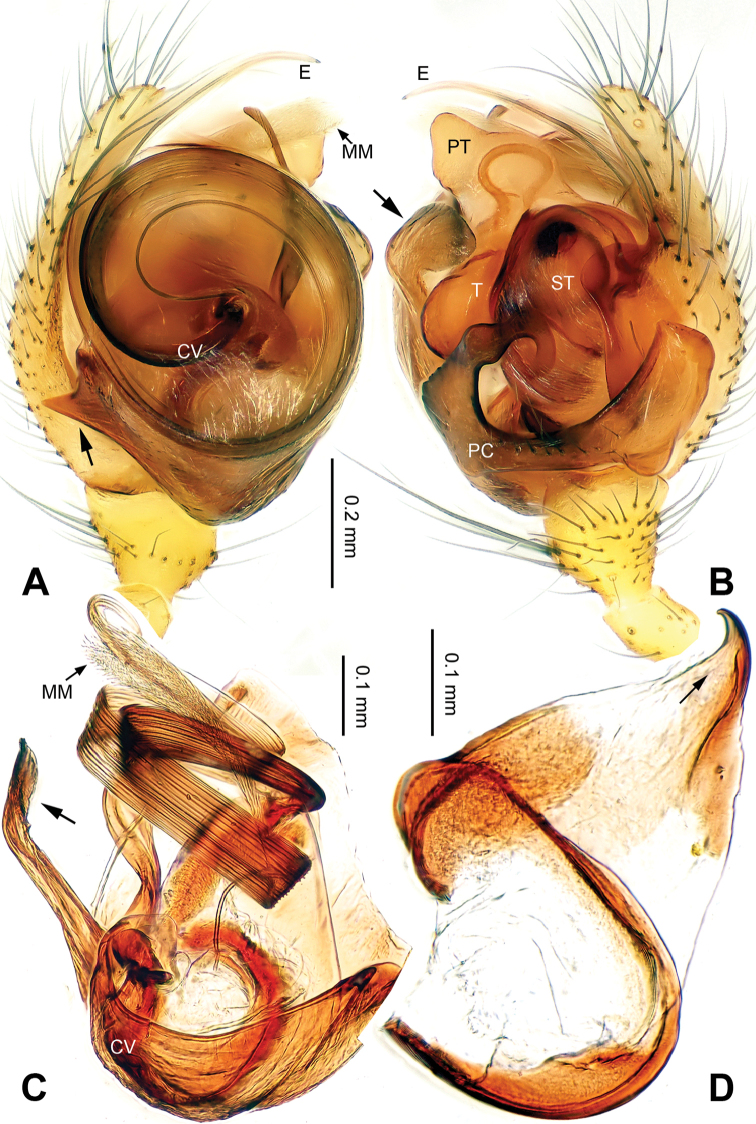
*Callosa baiseensis* sp. n., male holotype. **A** Palp, prolateral view **B** Palp, retrolateral view **C** Embolic division, retrolateral view **D** Distal suprategular apophysis, retrolateral view. Scale bars: **B** as **A**.

**Figure 6.**
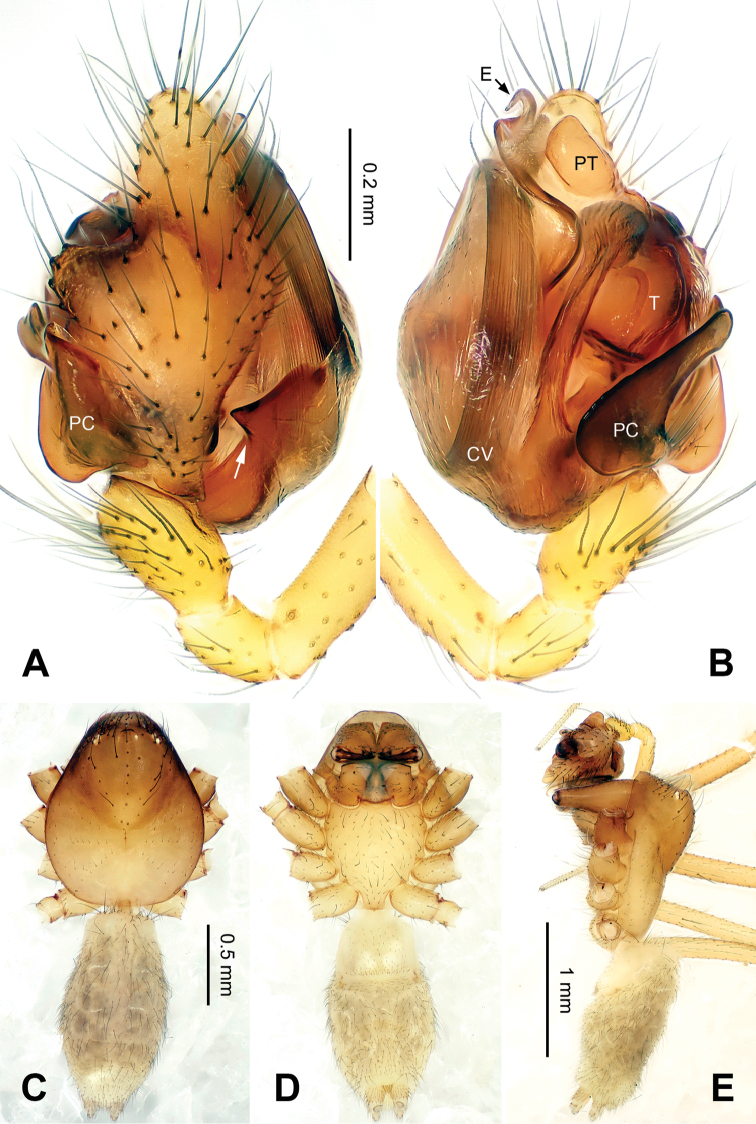
*Callosa baiseensis* sp. n., male holotype. **A** Palp, dorsal view **B** Palp, ventral view **C** Habitus, dorsal view **D** Habitus, ventral view **E** Habitus, lateral view. Scale bars: **B** as **A**; **D** as **C**.

*Male palp*: femur about four times longer than patella; tibia with two trichobothria, one ventral and one retrolateral (Fig. 5B). Cymbium spindle-shaped at dorsal view (Figs 2A, 6A); Paracymbium ‘J’-shaped, stout at base, attenuated and curved at apex (Figs 1B, 5B). Bulb with an oblate subtegulum and a protruding protegulum (Figs 1B, 5B). Convector with a membranous plate enveloping the prolateral side of embolic division (Figs 1A, 5A), and a ribbon-like ventral process (Figs 1B, 2B, 5B, 6B); dorsal projection of convector situated near the base of cymbium in prolateral view (Figs 1A, 5A); distal suprategular apophysis pick-like, broad at base, hooked at apex (Figs 1D, 5D); median membrane with dense membranous short cilia (Figs 4B, 8B); embolus long and belt-like, with a tapering tip, making 1.5 loops along the exterior margin of convector plate (Figs 1A, 5A).

**Figure 7.**
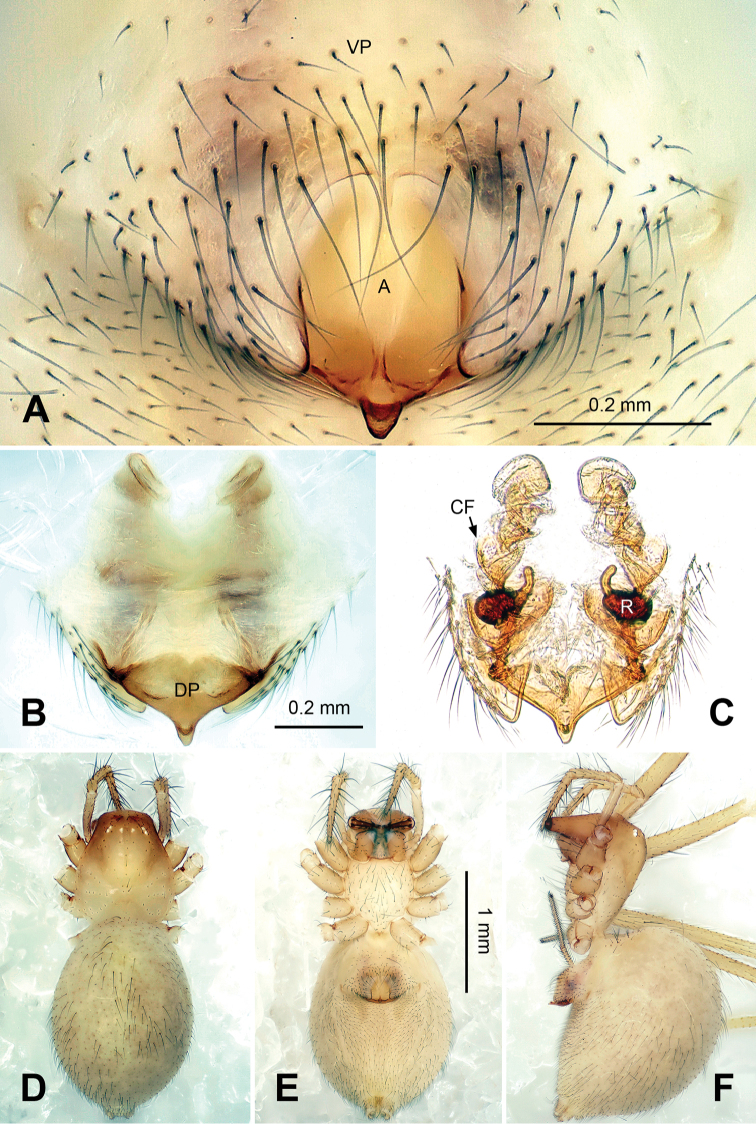
*Callosa baiseensis* sp. n., female paratype. **A** Epigyne, ventral view **B** Epigyne, dorsal view **C** Vulva, dorsal view **D** Habitus, dorsal view **E** Habitus, ventral view **F** Habitus lateral view. Scale bars: **C** as **B**; **D, F** as **E**.

**Figure 8.**
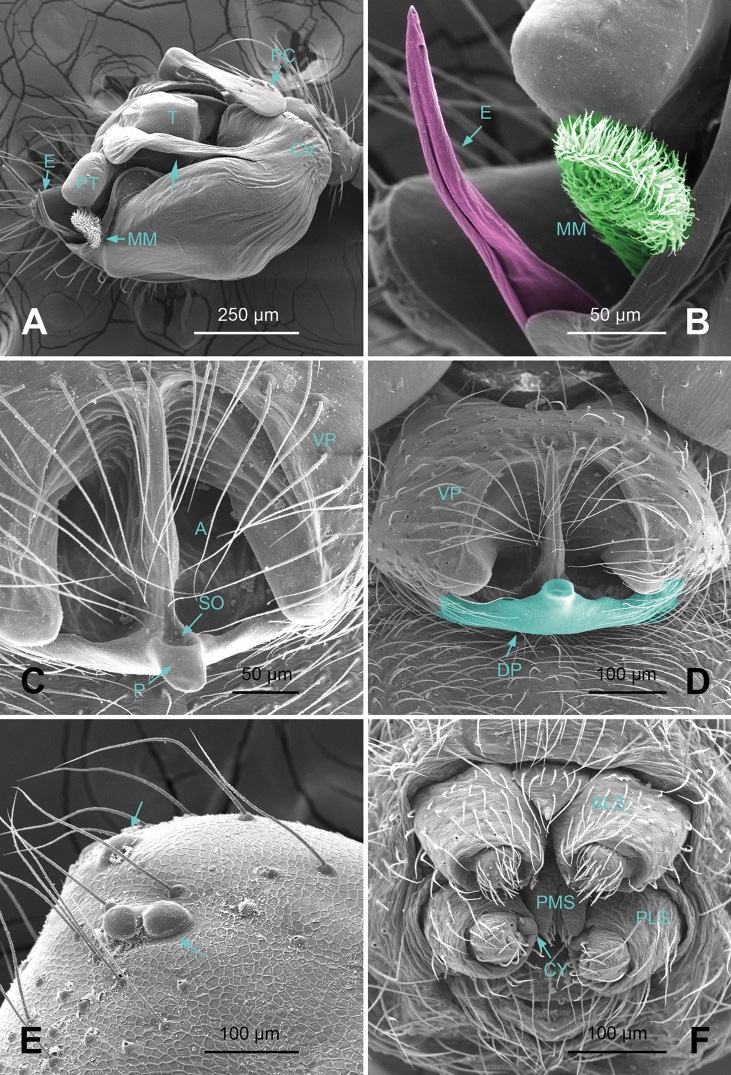
*Callosa baiseensis* sp. n., SEM of a male paratype and a female paratype. **A** Palp of male paratype, ventral view **B** Detail showing embolus and embolic membrane **C** Detail showing scape of epigyne **D** Epigyne of female paratype, ventral view **E** Anterior lateral eye, anterior median eye and posterior lateral eye of male paratype **F** Spinnerets of female paratype.

*Epigyne*: dome-shaped in lateral view, with atrium fully exposed in ventral view (Figs 3A, 4C–D, 7A, 8C–D); septum stretched along the axis of atrium; parmula short with a shallow socket near tip (Figs 4D, 8C); copulatory furrows making a spiral course (Figs 3C, 7C); receptacles oval, with short, tube-like processes (Figs 3C, 7C).

##### Species composition.

Two species, *Callosa ciliata* sp. n. (type species) and *Callosa baiseensis* sp. n.

##### Distribution.

**Figure 9.**
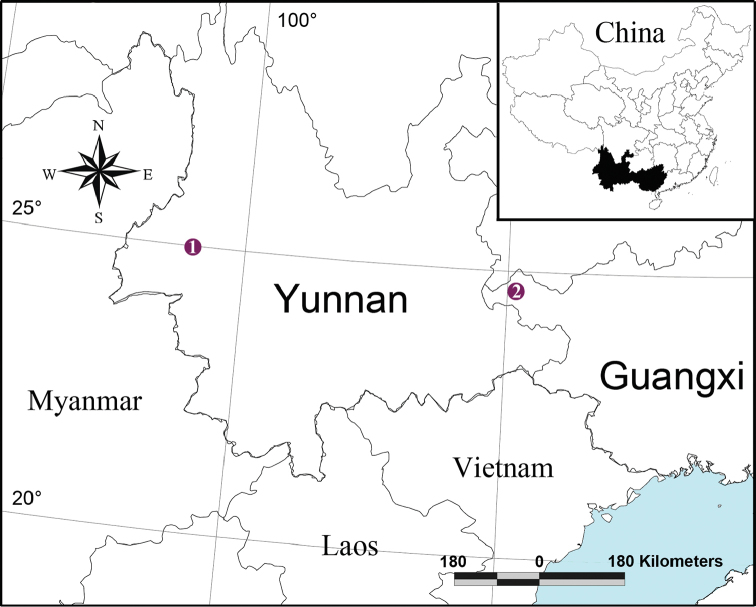
Type localities of new species *Callosa ciliata* sp. n. (**1**) and *C. baiseensis* sp. n. (**2**).

Yunnan Province and Guangxi Zhuang Autonomous Region, China (Fig. 9).

#### Callosa ciliata sp. n.

Taxon classificationAnimaliaAraneaeLinyphiidae

http://zoobank.org/2FF3B2E8-915E-487E-8F79-C5609A12D972

Figs 1 , 2 , 3 , 4 , 9

##### Types.

Holotype ♂: CHINA, Yunnan Province: Baoshan City: Tengchong County; Gudong Town; Jiangdong Village; 24°58.103'N, 98°52.104'E, ca 1900 m, Jiangdong Mountain, Luoshui Cave, 26.XI.2013, (Y.C. Li & J.C. Liu). Paratypes: 1♂ 2♀, same data as for holotype.

##### Etymology.

This specific name is taken from the Latin word ‘ciliatus’, meaning ‘with cilia’, which refers to the median membrane with cilia; adjective.

##### Diagnosis.

It is characterised by the subdivided tip of distal suprategular apophysis (Fig. 1D) and in having three coils in copulatory furrows in epigyne (Fig. 3C). *Callosa ciliata* sp. n. also has a narrower atrium and shorter parmula.

##### Description.

*Male (holotype)*. Total length: 2.60. Carapace 1.25 long, 0.94 wide, brownish yellow (Fig. 2C, E), AME and PME entirely lost, ALE and PLE strongly reduced (Figs 2E, 4E). Sternum 0.68 long, 0.63 wide. Clypeus 0.50 high. Eye sizes: ALE 0.02, PLE 0.03. Leg length: I 8.06 (2.10, 0.40, 2.38, 2.05, 1.13), II 7.44 (2.00, 0.38, 2.13, 1.88, 1.05), III 5.74 (1.56, 0.30, 1.50, 1.55, 0.83), IV 6.98 (2.03, 0.31, 2.03, 1.75, 0.86). TmI 0.20. Abdomen pale, with irregular dark patterns (Fig. 2C–E). Palp: paracymbium large, with distal end strongly curved inward; tegulum broad at base, protegulum conical, crooked at tip; distal suprategular apophysis with a small indentation at apex (Fig. 1D); convector with a sharp projection at the 8 o’clock position at prolateral view (Fig. 1A); convector’s ventral process ribbon-like, with a slightly broadened tip (Fig. 1B); embolus coiling from 4 o’clock position in prolateral view (Fig. 1A).

*Female*. Total length: 2.80. Carapace 1.25 long, 0.59 wide, same coloration as in male, AME vanished, ALE, PLE and PME reduced to white spots (Fig. 3D, F). Sternum 0.63 long, 0.69 wide. Clypeus 0.34 high. Eye sizes: ALE 0.03, PME 0.02, PLE 0.02. Leg length: I 8.21 (2.25, 0.40, 2.43, 2.00, 1.13), II 7.52 (2.18, 0.40, 2.19, 1.75, 1.00), III 5.79 (1.70, 0.38, 1.55, 1.38, 0.78), IV 7.07 (2.13, 0.35, 2.00, 1.75, 0.84). TmI 0.15. Abdomen with same coloration as in male (Fig. 3D, F). Epigyne: atrium roughly triangular in form, broad at posterior, narrowing towards anterior (Fig. 3A); fovea large, with ridged inner walls; parmula small; receptacles suboval, with digit-like outgrowth, separated by 3 diameters (Fig. 3C); copulatory furrows making 3 coils.

#### Callosa baiseensis sp. n.

Taxon classificationAnimaliaAraneaeLinyphiidae

http://zoobank.org/2433C26A-75D0-4B76-9720-1AA133CA168D

Figs 5 , 6 , 7 , 8 , 9

##### Types.

Holotype ♂: CHINA, Guangxi Zhuang Autonomous Region: Baise City; Longlin County; De’e Town; Yakou Village: 24°39.130'N, 105°09.557'E, ca 1500 m, Da Cave, 14–15.XII.2012, (Z.G. Chen & Z. Zhao). Paratypes: 1♂ 2♀, same data as for holotype; 1♀, Yumigan Cave, 24°39.145'N, 105°09.430'E, ca 1549 m, 14–15.XII.2012, (Z.G. Chen & Z. Zhao).

##### Etymology.

This specific name is derived from Chinese Pinyin ‘bǎi sè’ (), referring to its type locality; adjective.

##### Diagnosis.

Non-indented apex of distal suprategular apophysis (Fig. 5D), and the broad tip of convector ventral process in male palp (Figs 5B, 6B); it differs from the type species *C. ciliata* sp. n. by the relatively longer parmula (Figs 7B, 8C) and wider atrium (Fig. 7C).

##### Description.

*Male (holotype)*. Total length: 2.60. Carapace 1.20 long, 1.00 wide, beige, ocular area brownish yellow (Fig. 6C), AME completely lost, ALE, PLE and PME strongly reduced (Fig. 6C, E). Sternum 0.68 long, 0.66 wide. Clypeus 0.44 high. Eye sizes: ALE 0.03, PME 0.02, PLE 0.04. Leg length: I 9.25 (2.50, 0.38, 2.80, 2.41, 1.16), II 8.27 (2.28, 0.38, 2.38, 2.23, 1.00), III 6.33 (1.84, 0.40, 1.68, 1.56, 0.85), IV 8.05 (2.38, 0.38, 2.33, 2.03, 0.93). TmI 0.16. Abdomen pale, with dark yellow markings (Fig. 6C–E). Male palp: protegulum medially expanded, then attenuated at tip (Fig. 5B); distal suprategular apophysis with a small, hooked apex (Fig. 5D); embolus coiling from 8 o’clock position in prolateral view (Fig. 5C).

*Female*. Total length: 2.50. Carapace 1.19 long, 0.94 wide, same coloration as in male. Sternum 0.55 long, 0.63 wide. Clypeus 0.34 high. Eye sizes: ALE 0.05, PME 0.04, PLE 0.05. Leg length I 8.91 (2.48, 0.40, 2.56, 2.34, 1.13), II 8.30 (2.28, 0.40, 2.34, 2.19, 1.09), III 6.29 (1.88, 0.38, 1.63, 1.59, 0.81), IV 7.91 (2.30, 0.38, 2.15, 2.08, 1.00). TmI 0.18. Abdomen with same coloration as in male (Fig. 7D–E). Epigyne: atrium nearly semicircular, partitioned by a septum along the long axis (Fig. 8C–D); copulatory furrows forming 2 coils; receptacles oval separated by 2 diameters, with curved outgrowths (Fig. 7C–D).

##### Remarks.

To confirm the species delimitation, the p-distance of COI sequences of *C. baiseensis* sp. n. and *C. ciliata* sp. n. was calculated using MEGA 6 (Tamura et al. 2013), and the result is 0.12, which falls within the genetic distance interval of 0.07 to 0.16 among *Bathyphantes* species and 0.07 to 0.17 in *Porrhomma* based on data from NCBI (The National Center for Biotechnology Information https://www.ncbi.nlm.nih.gov/).

## Discussion

Linyphiidae Blackwall, 1859 is not commonly found in caves. In China, in contrast to more than 370 terrestrial linyphiids, only two species have been reported from caves so far (Song and Li 2009), but none of them exhibited traits of cave adaptation, such as depigmentation, reduction or complete loss of eyes, or elongation of legs (Sket 2008). *Callosa* gen. n. is the first true troglobiont linyphiid genus discovered in southwest China, encompassing two new species found in caves almost 600 kilometers apart, and they display apparent characters of true cave dwellers. It is assumed their ancestors were widely distributed in the montane area in southwest China, and almost certainly extrinsic forces (e.g. geological events, climatic changes) drove them to colonize the caves, which are considered to be a relatively stable environment.

**Figure 10.**
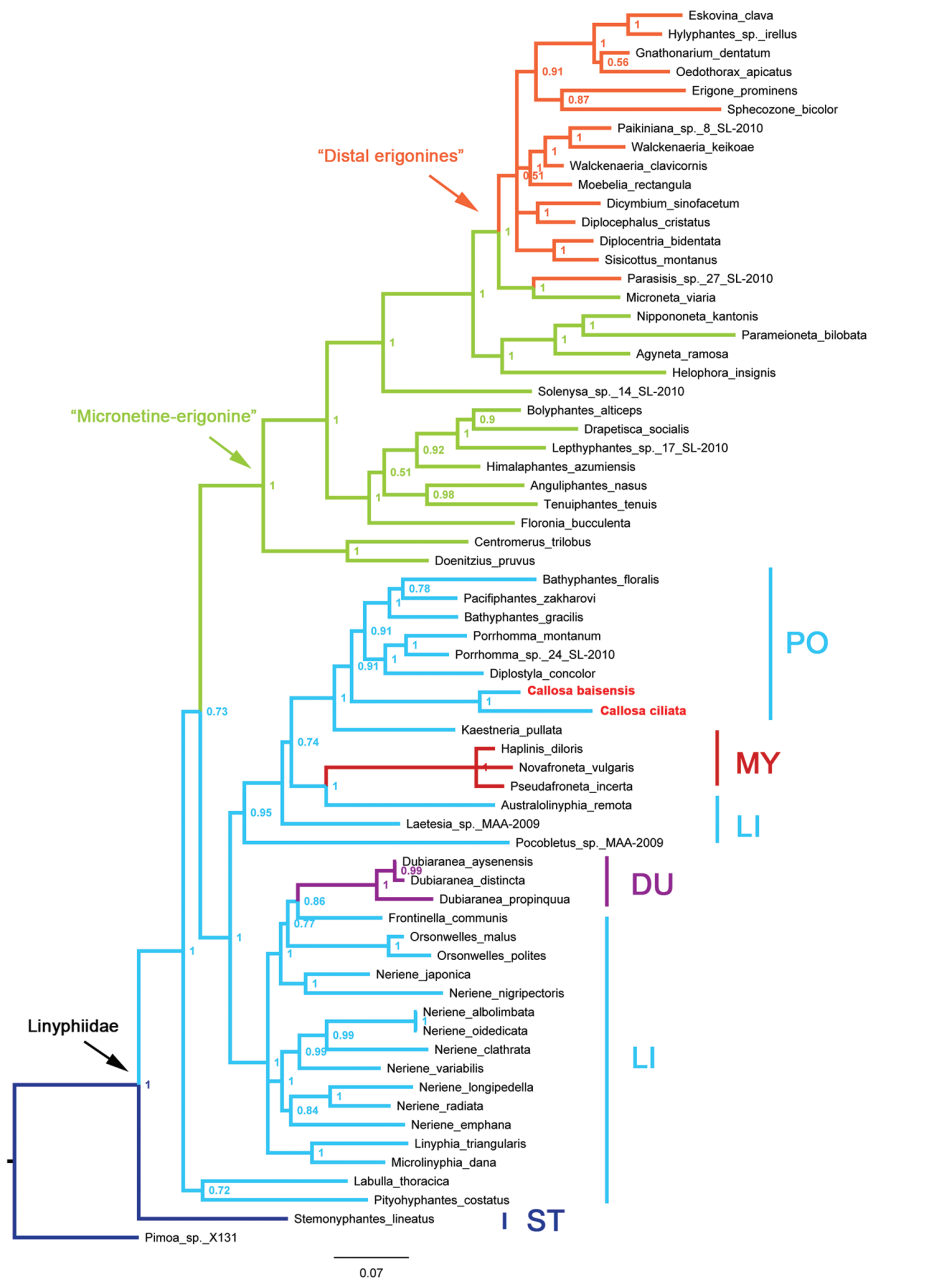
Phylogenetic tree reconstructed using Bayesian inference based on concatenated data. Numbers besides each node are posterior possibilities. Outgroup: *Pimoa* sp. X131 (dark blue) **DU** Dubiaraneinae (purple) **LI** Linyphiinae (blue) **MY** Mynogleninae (red) **PO** (blue) **ST** Stemonyphantinae (dark blue). “Micronetines-erigonines” clade is presented in green, the “distal erigonines” clade is colored in orange. Taxa with sequences downloaded from NCBI are listed at the end of each branch in black accordingly, and *Callosa* gen. n. species are marked in red.

*Callosa* gen. n. belongs to as suggested by both molecular analysis (Fig. 10) and morphological characteristics. It is obviously monophyletic, and its distinctive traits in both body and copulatory organs might be a result of long-term solitary evolution. Despite its morphological similarities to *Bathyphantes* (especially *B. approximatus*), *Callosa* gen. n. is situated relatively farther from *Bathyphantes* in the cladogram (Fig. 10). The taxonomical history of *Bathyphantes* is long and complicated, and several of its subgenera have now been validated as separate genera (e.g. *Kaestneria*, *Diplostyla*, *Pacifiphantes*) based on the conformation of copulatory organs, and some related genera were also established with species transferred from *Bathyphantes* (e.g. *Cresmatoneta* Simon, 1929, *Microbathyphantes* Helsdingen, 1985). A better-sampled phylogenetic analysis of was presented by Wang et al. (2015), in which *Bathyphantes* appeared as polyphyletic, with *Pacifiphantes zakharovi* Eskov & Marusik, 1994 grouped with *Bathyphantes eumenis* (L. Koch, 1879). The split between *Porrhomma* + *Diplostyla* and *Bathyphantes* is not well supported. A similar relationship is recovered in our analysis, where *Pacifiphantes zakharovi* is clustered with *Bathyphantes floralis* Tu & Li, 2006 (Fig. 10). It also has been previously pointed out that *Pacifiphantes magnificus* (Chamberlin & Ivie, 1943) could be a misplacement, and probably grouped with *Porrhomma* + *Diplostyla* as indicated by both morphology and DNA barcoding (Slowik and Blagoev 2012). As the type species, *Pacifiphantes zakharovi* was identified with a super short embolus (Eskov and Marusik 1994: fig. 42), the unique trait supposedly distinguishing it from other similar *Bathyphantes*, however, the discrepancy between morphology and molecular analysis results demands a more comprehensive analysis on the delimitation of *Bathyphantes* and its close relatives.

## Supplementary Material

XML Treatment for Callosa

XML Treatment for Callosa ciliata

XML Treatment for Callosa baiseensis

## References

[B1] Arnedo MA Hormiga G Scharff N (2009) Higher-level phylogenetics of linyphiid spiders (Araneae, Linyphiidae) based on morphological and molecular evidence. Cladistics 25: 231–262. https://doi.org/10.1111/j.1096-0031.2009.00249.x 10.1111/j.1096-0031.2009.00249.x 34879614

[B2] Eskov KY Marusik YM (1994) New data on the taxonomy and faunistics of North Asian linyphiid spiders (Aranei Linyphiidae). Arthropoda Selecta 2(4): 41–79.

[B3] Hall TA (1999) Bioedit: a user-friendly biological sequence alignment editor and analysis program for Windows 95/98/NT. Nucleic Acids Symposium Series 41: 95–98.

[B4] Ivie W (1969) North American spiders of the genus *Bathyphantes* (Araneae, Linyphiidae). American Museum Novitates 2364: 1–70.

[B5] Posada D (2008) jModelTest: phylogenetic model averaging. Molecular Biology and Evolution 25: 1253–1256. https://doi.org/10.1093/molbev/msn083 18397919 10.1093/molbev/msn083

[B6] Roberts MJ (1987) The spiders of great Britain and Ireland. Volume 2. Harley Books, England, 204 pp.

[B7] Ronquist F Huelsenbeck JP (2003) MrBayes 3: Bayesian phylogenetic inference under mixed models. Bioinformatics 19(12): 1572–1574. https://doi.org/10.1093/bioinformatics/btg180 12912839 10.1093/bioinformatics/btg180

[B8] Saaristo MI (1995) Linyphiid spiders of the granitic islands of Seychelles (Araneae, Linyphiidae). Phelsuma 3: 41–52.

[B9] Sket B (2008) Can we agree on an ecological classification of subterranean animals? Journal of Natural History 42: 1549–1563. https://doi.org/10.3956/2007-55.1

[B10] Slowik J Blagoev GA (2012) First description of the male spider *Pacifiphantes magnificus* (Chamberlin & Ivie) (Araneae: Linyphiidae). Zootaxa 3481: 73–81.

[B11] Song YJ Li SQ (2009) Two new erigonine species (Araneae: Linyphiidae) from caves in China. The Pan-pacific Entomologist 85(2): 58–69. https://doi.org/10.3956/2007-55.1

[B12] Sun N Marusik YM Tu L (2014) *Acanoides* gen. n., a new spider genus from China with a note on the taxonomic status of Acanthoneta Eskov & Marusik, 1992 (Araneae, Linyphiidae, Micronetinae). ZooKeys 375: 75–99. https://doi.org/10.3897/zookeys.375.6116 10.3897/zookeys.375.6116 PMC3921563 24526845

[B13] Tamura K Stecher G Peterson D Filipski A Kumar S (2013) MEGA6: Molecular Evolutionary Genetics Analysis Version 6.0. Molecular Biology and Evolution 30: 2725–2729. https://doi.org/10.1093/molbev/mst197 24132122 10.1093/molbev/mst197 PMC3840312

[B14] Tanasevitch AV (2014) New species and records of linyphiid spiders from Laos (Araneae, Linyphiidae). Zootaxa 3841(1): 67–89. https://doi.org/10.11646/zootaxa.3841.1.3 25082028 10.11646/zootaxa.3841.1.3

[B15] Tu LH Li SQ (2006) Three new and four newly recorded species of Linyphiinae and Micronetinae spiders (Araneae: Linyphiidae) from northern Vietnam. The Raffles Bulletin of Zoology 54: 103–117.

[B16] Wang F Ballesteros JA Hormiga G Chesters D Zhang YJ Sun N Zhu CD Chen W Tu LH (2015) Resolving the phylogeny of a speciose spider group, the family Linyphiidae (Araneae). Molecular Phylogenetics and Evolution 91: 135–149. https://doi.org/10.1016/j.ympev.2015.05.005 25988404 10.1016/j.ympev.2015.05.005

